# Experimental infection of cotton rats and bobwhite quail with *Rickettsia parkeri*

**DOI:** 10.1186/1756-3305-6-70

**Published:** 2013-03-15

**Authors:** Gail Miriam Moraru, Jerome Goddard, Christopher D Paddock, Andrea Varela-Stokes

**Affiliations:** 1 Department of Basic Sciences, College of Veterinary Medicine, Mississippi State University, Wise Center, Spring Street, Mississippi State, MS, 39762, USA; 2 Department of Biochemistry, Molecular Biology, Entomology and Plant Pathology, Mississippi State University, Mississippi State, MS, USA; 3 Infectious Diseases Pathology Branch, Centers for Disease Control and Prevention, Atlanta, GA, USA

**Keywords:** Rickettsia parkeri, Experimental infection, Cotton rat, Quail

## Abstract

**Background:**

*Amblyomma maculatum* is the primary vector for *Rickettsia parkeri*, a spotted fever group rickettsia (SFGR) and human pathogen. Cotton rats and quail are known hosts for larval and nymphal *A. maculatum*; however, the role of these hosts in the ecology of *R. parkeri* is unknown.

**Methods:**

Cotton rats and quail were inoculated with low or high doses of *R. parkeri* (strain Portsmouth) grown in Vero cells to evaluate infection by *R. parkeri* in these two hosts species. Animals were euthanized 2, 4, 7, 10, and 14 days post-injection (dpi) and blood, skin, and spleen samples were collected to analyze by Vero cell culture and polymerase chain reaction (PCR). In a second trial, cotton rats and quail were inoculated with *R. parkeri* and nymphal *A. maculatum* ticks were allowed to feed on animals. Animals were euthanized on 14, 20, 28, 31, and 38 dpi and blood and tissues were collected for serology and PCR assays. Fed ticks were tested for *R. parkeri* by PCR and Vero cell culture.

**Results:**

*Rickettsia parkeri* was isolated in cell culture and detected by PCR in skin, blood, and spleen tissues of cotton rats in the initial trial 2, 4, and 7 dpi, but not in quail tissues. In the second trial, no ticks tested positive for *R. parkeri* by PCR or cell culture.

**Conclusions:**

These studies demonstrate that viable *R. parkeri* rickettsiae can persist in the tissues of cotton rats for at least 7 days following subcutaneous inoculation of these bacteria; however, quail are apparently resistant to infection. *Rickettsia parkeri* was not detected in nymphal ticks that fed on *R. parkeri*-inoculated cotton rats or quail, suggesting an alternate route of transmission to naïve ticks.

## Background

Spotted fever group rickettsiae (SFGR) are vector-borne organisms often causing disease in humans. *Rickettsia rickettsii*, the causative agent of Rocky Mountain spotted fever (RMSF), is the best studied and most virulent of the SFGR [1]. The pathogenic potential of many other SFGR, however, is not well-documented, particularly for recently recognized rickettsial species. Further, while the genetic relatedness of known and emerging rickettsiae [2-4] and their presence in certain animal populations [5-7] have been described, basic ecology and epidemiology are less well-understood.

Despite the initial recognition of *R. parkeri* in 1937 [8], studies of this SFGR only increased substantially after 2004, when the first case of human infection was reported [9]. Subsequent seroprevalence surveys demonstrated certain animal species, including opossums, capybaras, and dogs, to be naturally exposed to *R. parkeri*, or a closely related SFGR [10-12].

Our understanding of the natural history of *R. parkeri* is mainly limited to its occurrence in the primary tick vector, *Amblyomma maculatum*, commonly known as the Gulf Coast tick. *Rickettsia parkeri* has been detected in 12%-43% of questing adult Gulf Coast ticks collected across the southeastern United States [13-15], suggesting this *Rickettsia* species is efficiently transmitted from the nymphal stage to the adult stage. It is unknown, however, if larval and nymphal *A. maculatum* acquire the microorganism predominantly by feeding on rickettsemic vertebrate hosts, through effective transovarial and transstadial transmission, or a combination of these transmission routes. Both larval and nymphal Gulf Coast ticks feed on small mammals such as cotton rats and ground-dwelling birds, including meadowlarks and northern bobwhite [16-18]. Adult stages parasitize larger mammals including cattle, goats, deer, dogs, and occasionally humans [19]. Experimental infection studies showed that opossums (*Didelphis aurita*) and cattle seroconverted when inoculated with *R. parkeri*. Some animals (2/6 calves and 1/2 opossums) also became transiently rickettsemic [20,21]. It is not known, however, if one or more vertebrate hosts act as reservoirs or amplifying hosts for *R. parkeri*, as described previously for *R. rickettsii*, the agent of RMSF [22-24].

This study was performed to assess the infectivity of *R. parkeri* to cotton rats and bobwhite quail, two recognized vertebrate hosts for larval and nymphal stages of *A. maculatum*, and to investigate the ability of nymphal ticks to acquire *R. parkeri* from these *R. parkeri*-exposed hosts.

## Methods

### Animal and tick sources

Cotton rats (*Sigmodon hispidus*) were purchased from Harlan Laboratories (Indianapolis, IN). Northern bobwhite quail were purchased from P & L Crowley Farm (Maben, MS).

Ticks were purchased from Texas A&M University (TAMU) and Oklahoma State University (OSU). Those from the latter institution, have previously been found to be infected with “*Candidatus* Rickettsia andeanae” (100% of those tested), while ticks from the TAMU colony are not known to be positive for this organism (Moraru, unpublished data). DNA was extracted individually from nymphal ticks obtained from both institutions and PCR amplified using primers 16S+2 and 16S-1 to target the 16S rDNA gene as confirmation that tick DNA had been extracted [25]. Extractions were then tested by PCR amplification targeting SFGR-wide *rompA* and “*Ca.* R. andeanae”-specific *rompA* gene fragments. The former was an assay using primers 190–70 and 190–701 for the primary reaction and primers 190-FN1 and 190-RN1 for the secondary reaction [26]; the latter used primers Rx-190-F and Rx-190-R [14].

### Culture for injections

*Rickettsia parkeri* was grown in Vero cell culture with minimum essential media (MEM with Earle’s salts) supplemented with 10% fetal bovine serum. A low passage (P4 and P5) isolate of *R. parkeri* (Portsmouth) was used for all animal infections. Infected cultures were harvested when at least 90 percent of the Vero cells were infected, as determined by cell counts using 50 μl in a hemocytometer.

### Experimental exposure

The initial trial consisted of eleven quail and eleven cotton rats. All animals were pre-screened for SFGR antibodies via immunofluorescent antibody (IFA) testing (described in detail below). Five quail and five cotton rats received low dose injections of *R. parkeri* (1000 infected Vero cells in 0.2 ml of culture media). Another set of five quail and five cotton rats were injected with a high dose of the organism (10 000 infected Vero cells in 0.2 ml). Percent infectivity of Vero cells was estimated by cytospin, and cell counts were performed using a hemocytometer. Animals were injected subcutaneously, at the nape of the neck on cotton rats and in the right leg of quail. One individual of each species served as a negative control and was injected with 10 000 uninfected Vero cells in a 0.2 ml volume. Four out of twenty animals—one low dose quail, one low dose rat, one high dose quail, and one high dose rat—were numbered and randomly selected from each group for euthanasia at 2, 4, 7, 10, and 14 days post injection (dpi). The controls were euthanized on 14 dpi. Animals to be euthanized were numbered and selected at random, within their dose assignment, and euthanized using carbon dioxide.

Upon euthanasia, blood was collected from the animals via intracardiac puncture. A 250 μl volume of whole blood from each animal was placed into individual flasks of confluent Vero cells. Skin from the injection site and spleen tissue samples were collected on necropsy. Half of each tissue sample was put into Vero cell culture (described in cell culture section below), and half was frozen at −20°C until DNA extractions and PCR assays could be performed.

### Experimental tick infestation

The second trial consisted of eleven cotton rats and eleven quail. One cotton rat and one quail were injected with 10 000 uninfected Vero cells in 0.2 ml of culture media. The remaining ten individuals of each species received injections of *R. parkeri* infected Vero cell culture (10 000 cells in 0.2 ml each). At 4 dpi, nymphal *A. maculatum* ticks were placed on each of the animals (as larvae, ticks from OSU were fed on rabbits and TAMU ticks on chickens). Two cotton rats and two quail had OSU ticks (n=50), while all remaining animals (including controls) received TAMU ticks (n=65). Trays underneath each animal’s cage were examined daily for detached engorged ticks. All detached ticks were placed in humidity chambers (90% RH) and allowed to molt. Ticks were allowed to feed for 13 days, after which a blood sample was taken from the animals for IFA testing (dpi 17).

Animals were euthanized on dpi 20, 24, 31, and 38. Upon euthanasia, a blood sample was collected via intracardiac puncture. At necropsy, tissues including skin from the original injection site, liver, spleen, kidney, and scrotal tissue (male rats only) were collected and stored at −20°C until DNA extraction and PCR testing could be performed. All experiments were approved by the Institutional Animal Care and Use Committee at Mississippi State University (IACUC 10–067).

### Indirect Fluorescent Antibody (IFA) test

Plasma from the blood samples was used to determine if SFGR IgG antibodies were present. Samples were screened at a 1:64 dilution. A 1:60 dilution of fluorescein isothiocyanate (FITC) anti-rat IgG (H+L) (KPL, Gaithersburg, MD) was used as a secondary antibody for rat samples; FITC anti-chicken (H+L) (KPL, Gaithersburg, MD) was used for the quail at a dilution of 1:275. Cotton rats and bobwhite quail known from previous IFA screening studies to be seronegative or seropositive for SFGR were used as controls.

### Cell culture of vertebrate tissues and ticks

Tissues, of approximately 1cm^2^, from animals in the first trial were triturated, using a sterile scalpel blade, into 250 μl of MEM + 10% FBS and added to 25 cm^2^ flasks of Vero cell culture. All flasks (3 different tissues per individual animal) received 10 μl penicillin-streptomycin (10000 U/ml penicillin and 10 mg/ml streptomycin).

In the second trial, ticks that successfully molted after feeding on *R. parkeri*-inoculated animals were pooled from each individual host. For example, all ticks that fed on quail 1 were put into one culture flask. Ticks were prepared for culture following a previously described protocol [27]. Briefly, they were put through a series of disinfecting washes. Each pool of ticks was placed into a 15 ml tube with 10 ml of a wash solution. For each wash, the tubes were vortexed for 3 min, after which the liquid was aspirated out. Washes were, in order: hydrogen peroxide, 70% ethanol, 20% household bleach, and sterile PBS. After this series of washes, ticks were cut using a sterile scalpel blade in a sterile petri dish, one at a time. Each tick was placed onto 0.2 ml cell culture media and bisected longitudinally. One half was retained and placed at −20°C for subsequent DNA extraction and PCR testing. The other half was triturated in the media in the petri dish and then placed in a 25 cm^2^ culture flask along with any other triturated ticks that had fed on the same animal. Each flask also contained 100 μl of penicillin-streptomycin (10000 U/ml penicillin and 10 mg/ml streptomycin) and 5 μl of amphotericin B (250 μg/ml).

Two days after tissues and ticks were placed in culture, flasks were emptied and fresh media was added. Flasks were then monitored weekly for three to six weeks for infection using cytospin preparations and acridine orange staining. Briefly, slides were allowed to air-dry and then were placed in methanol for 10 min. Slides were then flooded with acridine orange for 2-3 min.

### DNA extractions

DNA was extracted from rodent blood samples using GE Healthcare’s illustra blood genomicPrep Mini Spin kit (GE Healthcare, Piscataway, NJ). DNA was extracted from quail blood using the QIAamp DNA Blood Midi kit (Qiagen Inc., Valencia, CA). In all cases, 50 μl of blood was used for extraction following the kit protocols, as supplied by the manufacturer.

DNA was extracted from cell culture and ticks halves using GE Healthcare’s illustra tissue and cells genomic Prep Mini Spin kit and following the manufacturer’s protocols (GE Healthcare, Piscataway, NJ). A 200 μl volume was extracted from all harvested cultures. Tick halves were triturated into extraction buffer using a new sterile scalpel blade for each individual sample. Final elution volumes were 200 μl.

### Polymerase chain reaction (PCR)

A nested PCR program targeting a segment of the rickettsial outer membrane protein A (*rompA*) gene was used with primers 190–70 and 190–701 for the primary reaction and primers 190-FN1 and 190-RN1 for the secondary reaction [26]. *Rickettsia parkeri* DNA extracts (Portsmouth) and non-template water controls were included in the assays.

To test tick samples, this *rompA* PCR was preceded by a reaction using primers 16S+2 and 16S-1 to target the 16S rDNA gene as confirmation that tick DNA was extracted [25]. Ticks were tested with primers Rx-190-F and Rx-190-R, specific for “*Ca.* R. andeanae” and using a single reaction PCR assay [14].

## Results

### Rickettsia parkeri infection animals

The pre-inoculation serum samples obtained from all cotton rats and quail revealed no evidence of antibodies reactive with SFGR at a dilution of 1:64 or higher. Cotton rats and quail euthanized on dpi 2 and 4 were not seropositive; however, animals euthanized on dpi 7, 10, and 14 were seropositive. Control animals were seronegative. No blood samples tested positive by PCR at the time of euthanasia.

Results of PCR assays are shown in Table 1. Briefly, rickettsial DNA was detected in the skin sample of a cotton rat at dpi 4, and also in cultures of blood, skin, and spleen from cotton rats. *Rickettsia parkeri* was re-isolated from blood, skin, and spleen tissues from cotton rats, but not from any quail tissues.

**Table 1:** **PCR results from experimental infection with *R. parkeri *in cotton rats via injection**

	**dpi 2**		**dpi 4**		**dpi 7**		**dpi 10**		**dpi 14**	
	**Low**	**High**	**Low**	**High**	**Low**	**High**	**Low**	**High**	**Low**	**High**
Skin	- / +*	- / +	- / +	+ / +	- / +	- / -	- / -	- / -	- / -	- / -
Blood	- / -	- / -	- / +	- / +	- / -	- / -	- / -	- / -	- / -	- / -
Spleen	- / -	- / -	- / -	- / +	- / -	- / -	- / -	- / -	- / -	- / -

Tissues were PCR tested and placed in Vero cell culture. “Low” and “high” indicate the dose the animal received (10000 or 100000 infected *R. parkeri* Vero cells). Days post injection (DPI) across the table represent time of euthanasia and tissue collection.

* Signs before the slash indicate tissue results prior to culture, and signs after the slash signify PCR results of tissues after culture.

### Experimental infections with ticks

In the series of experiments where *R. parkeri*-infected animals were exposed to nymphal *A. maculatum*, 2 *R. parkeri*-exposed rats died, due to undetermined causes before completion of the tick feeding period (one on dpi 13 and one on dpi 15). Time of death allowed for blood to be obtained from only one of these rats. The control rat died on dpi 24, also from unknown causes apparently unrelated to the study; blood and spleen samples were collected and tested by PCR and found to be negative for SFGR DNA.

All animals were seronegative on pre-screen by IFA. Controls remained seronegative throughout the study. On 17 dpi, 8 of 9 rats and 5 of 10 quail were positive for SFGR antibodies at a 1:64 dilution. No blood samples at this time-point tested positive by PCR. Two quails (one sampled 31dpi and the other on 38 dpi) were seropositive (1:64) at the time of euthanasia. All rats were seropositive (1:64) at the time of euthanasia. No blood samples or other animal tissues tested positive by PCR at any time point.

A total of 61 engorged ticks were recovered from the quail (0–12) and cotton rats (0–8), representing a range of 0–7 ticks per animal and resulting in 13 culture flasks (1–7 ticks per animal). All cell cultures and ticks tested negative by PCR for rickettsial DNA. OSU ticks remained positive for “*Ca.* R. andeanae” after feeding on animals.

## Discussion

This study demonstrates that needle-inoculated cotton rats can maintain infection with *R. parkeri* in various tissues for at least 4–7 days. Nonetheless, *R. parkeri* appears to be rapidly cleared by the immune system of cotton rats and even more quickly in bobwhite quail. Previous work with cotton rats indicated that *R. rickettsii* was cleared within 24 hours of infection [23].

Although some rickettsiae have vertebrate reservoirs or amplifiers [28], in other cases, the tick vector is implicated as a reservoir of certain rickettsiae. *Rickettsia honei* occurred in 63% of *Aponomma hydrosauri* ticks collected (n=46), but not in lizard hosts (n=17) [29]. Infection rates of *A. maculatum* with *R. parkeri* are also high. In Virginia, rates of infection with *R. parkeri* greater than 40% in *A. maculatum* have been reported [15,30]. One study reports this rickettsia in 28% of *A. maculatum* ticks sampled in Mississippi and Florida, with a maximum infection rate of 40% in Jackson County, Mississippi [13]. Another study found a prevalence of 15.2% in *A. maculatum* collected throughout Mississippi [31]. Additionally, wild-caught rodents and bobwhite quail from farms in Mississippi have shown serological evidence of exposure to SFGR [32].

It appears that the ecology of *R. parkeri* is not dependent upon cotton rats or quail as reservoirs. While some feeding ticks may acquire the SFGR by feeding on recently infected animals such as cotton rats, there may be another mode of horizontal transmission occurring. Although our samples were small, our results suggest cotton rats and quail do not effectively transmit *R. parkeri* to naïve ticks. However, we also performed needle inoculations using cultured *R. parkeri* and laboratory-reared ticks, which may result in different infection dynamics than feeding naturally infected ticks on naïve animals. Transmission via co-feeding, from an infected tick to a naïve tick feeding nearby on the host, has been demonstrated with *R. massilae* and *R. conorii*[33,34], suggesting the tick vectors may be acting as reservoirs instead of the vertebrate hosts. This would mean a less important role for vertebrate hosts in terms of pathogen maintenance. This merits further investigation in the context of *R. parkeri*.

## Conclusions

This study adds valuable information to our limited knowledge of the dynamics of *R. parkeri* in avian and mammalian hosts. Cotton rats may serve briefly as sources of infection for feeding ticks, or there may be other vertebrate species that have this potential. While cotton rats did not show substantial evidence of *R. parkeri* circulating in blood, they may still have the potential to infect naïve *A. maculatum* ticks indirectly (co-feeding) or during acute infection. With the increasing number of recognized *R. parkeri* cases in humans, it is important to identify potential sources of infection. Many questions remain and the ecology of this tick-borne rickettsia still needs to be examined thoroughly, both with surveys of potential vertebrate reservoirs and through experimental studies.

## Competing interests

The authors declare that they have no competing interests.

## Authors’ contributions

GMM contributed to study design, laboratory work, intellectual interpretation, and writing of the manuscript. JG was involved in study design, intellectual interpretation, and revision of the manuscript. CDP contributed valuable intellectual content and revised the manuscript critically. AVS supervised and designed the study, contributed intellectual content, and performed critical revisions of the manuscript. All authors read and approved the final version of the manuscript.

## Authors’ information

The findings and conclusions are those of the authors and do not necessarily represent the official position of the Centers for Disease Control and Prevention.
